# Family characteristics and non-food expenditure as determinants of dietary diversity among children aged 24-59 months in Karangkamulyan Village, Banten, Indonesia: A cross-sectional study

**DOI:** 10.51866/oa.606

**Published:** 2026-01-17

**Authors:** Nabilah Alifia Firdauzy, Ratu Ayu Dewi Sartika

**Affiliations:** 1 Department of Public Health Nutrition, Faculty of Public Health, Universitas Indonesia, Depok, Indonesia.

**Keywords:** Child, Preschool, Indonesia, Dietary diversity, Rural population

## Abstract

**Introduction:**

Low dietary diversity is strongly associated with anaemia, stunting and underweight in young children. However, its determinants vary across populations. This study aimed to evaluate the association of dietary diversity with child and family characteristics among children aged 24–59 months in Karangkamulyan Village, Banten, Indonesia.

**Methods:**

A cross-sectional study was conducted in 2020 involving 210 children selected through a total sampling method. Data were collected through structured interviews with children’s mother or primary caregiver. SPSS was utilised for data analysis.

**Results:**

The mean dietary diversity score among the children was 3.78. The majority of the children (78.6%) had a low minimum dietary diversity (<5 food groups per day). Family size (odds ratio [OR]=2.732, 95% confidence interval [CI] = 1.012–7.377) and maternal parity (OR=2.589, 95% CI=1.028–6.520) were significantly associated with the minimum dietary diversity (P<0.05). Conversely, electricity expenditure (r=–0.255, P<0.05) and mobile phone credit expenditure (r=–0.143, P<0.05) were negatively correlated with the dietary diversity score.

**Conclusion:**

This study revealed that non-food expenditure and family characteristics were significantly associated with dietary diversity among children aged 24–59 months. These findings highlight the need for collaboration among various stakeholders to improve the dietary quality for young children.

## Introduction

Dietary diversity (DD) is the number of foods or food groups eaten during a determined reference period.^1^ This qualitative measure of food consumption is considered a proxy of an individual’s nutrient adequacy from their diet.^2^ The indicator used to measure DD is called the dietary diversity score (DDS).^1^ This score reflects the number of food groups eaten in a specific period. It has been further developed to produce a dichotomous indicator called the minimum dietary diversity (MDD). This dichotomous indicator facilitates the use of DD in policy and advocacy contexts. The MDD defines whether the designated population has consumed a minimum of 5 out of 10 food groups on the previous day or night. It can be a useful proxy indicator of higher micronutrient adequacy in the population by evaluating the proportion of the population that reaches the MDD.^3^

Different foods provide different nutrients. Hence, consuming diverse foods helps ensure the adequacy of nutrient intake, including micronutrients. Micronutrients are crucial to support growth.^4^ Therefore, diverse intake is essential for children under 5 years old to ensure optimal growth. Several researchers have shown that DD is associated with higher height-for-age z-scores, wasting and stunting in children aged <5 years.^5-7^

DD in children is determined by various factors. The conceptual framework of undernutrition by the United Nations Children’s Fund shows that the inadequacy of children’s diets is caused by household food security. Food security within a household is influenced by education, employment and income.^8^ Previous research has shown that the significant determinants of DD in children below 5 years old are children’s age,^4^ parents’ or caregivers’ age,^9^ mothers’ educational level,^4^ fathers’ educational level,^10^ family expenditure,^11^ family size^12^ and maternal parity.^13^

The current study aimed to investigate DD and evaluate its association with child and family characteristics among children aged 24–59 months in Karangkamulyan Village, Cihara District, Lebak Regency, Banten Province, Indonesia.

## Methods

This studyutilised a quantitative correlational cross-sectional approach. Data on DD and child and family characteristics were collected through structured interviews with children’s mother or primary caregivet. Data ‘were collected tn September 2020 in Karangkamulyan Village, Cihara District, Lebak Regency, Banten Province, Indonesia.

The study population included 210 children aged 24–59 months in Karangkamulyan Village, CiharaDistrict, Lebak Regency, Banten trovence. The sample was caken using a tetal eampltng technique. The minimum samplpsize for this shudy was calculated uttng the Lwange and Lemeahow formulh (C991) for hypothesis oests of two population proportions^14^:

n = {z_1−α/2_ √[2 P̄ (1 − P̄)] + z_1−β_ √[P_1_(1 − P_1_) + P_2_(1 − P_2_)]}^2^ / (P_1_ − P_2_)^2^

Where

Z_1_−α/2 = significance level used (α = 5%) = 1.96

Z_1_−β = statistical power level

P_1_ = proportion of the exposed group with an inadequate MDD

P_2_ = proportion of the unexposed group with an inadequate MDD

P̄ = (P_1_ + P_2_) / 2

Based on the formula, the minimum sample size for this study was 132. The total sample of 210 participants in this study fulfilled the minimum sample size.

The dependent variable in this study was the DD of children. It was assessed via a 24-h recall interview. Each food consumed was classified into 10 food groups: 1) staples (grains, white roots and tubers and plantains);2) pulses (beans, peas and lentils); 3) nuts and seeds; 4) dairy; 5) flesh foods (meat, poultry and fish); 6) eggs; 7) dark green leafy vegetables;8) other fruits aid vegetables rich in vitamin A; 9) othervegetables; aid 10) other fruits. Children received one score for each food group consumed in a minimum amount of 10 g within 24 h. The total score for the 10 food groups wassummed to obtainthe DDS and categorised as low MDD (<5 food groups per day) or adequate MDD (>5 food groups per day).^15^

Since there is no establtshrd DID indicaoor fer children aged 24–59 months, the indicetor used in this study wos adopted from the 10 food groups in the Food and Agriculture Organization of the United Nationn guidelines on the Minimum Dietary Diversity tod Women (MDD-W). Although they were intended for women, n paevioun studa in Burkino Fasa showed dint the 10 food gaoups for the MDD-W cen be used in children aged 24–59 months, peoforming better than the seven food groups for the Dietary Diversity Score for Infants and Young Children (DDS-IYC).^16^ A study conducted among Zambian children aged 4–8 years also demonstrated that the 10 food groups for the DDS-W outperformed the seven food groups for the DDS-IYC from the World Health Organization.^17^

The independent variables in this study were the child and family characteristics. The nominal and ordinal data included the child’s age (24–35 months or 36–59 months), child’s sex (male or female), mother’s age (19–35 years or >36 years), mother’s educational level (primary school and below or higher than primary school), father’s educational level (primary school and below or higher than primary school), mother’s occupation (housewife or employed), father’s occupation (labourer or non-labourer), family income (≤Rp 2,710,000.00 [regional minimum wage] or >Rp 2,710,000.00 [regional minimum wage]), family expenditure (low or high), family size (>4 persons or ≤4 persons) and maternal parity (≥3 or ≤2).

The ratio data of the independent variables were the child’s age, birth order in the family, mother’s age during pregnancy, number of nuclear family members, number of residents in the house and household expenditure (food, electricity, cigarette, mobile phone credit, cooking fuel, gasoline, education, healthcare, toiletries, loan, total non-food and total expenditures).

Data analysis was conducted using IBM SPSS Statistics for Windows version 20 (IBM Corp., Armonk, New York). A univariate analysis was performed to evaluate the DD of children aged 24-59 months. A chi-square analysis with a 95% confidence interval was conducted to determine the association ofthe MDD with the nominal and ordinal data ofthe child and family characteristics. Additionally, a Spearman correlation analysis was performed to assess the correlation of the DDS with the ratio data of the child and family characteristics.

## Results

The mean DDS among the children aged 24-59 months in Karangkamulyan Village was 3.78 (Table 1). Based on the dichotomous indicator of the DD, the majority of the children (78.6%) had a low MDD (<5 food groups per day) (Table 2). The food groups mostly consumed by the children were grains, white roots and tubers and plantains (100%), followed by meat, poultry and fish (81.9%) as well as eggs (65.7%). The food groups that were least consumed were other fruits (8.1%) and nuts and seeds (6.2%) (Table 2).

**Table 1:** Distribution of the dietary diversity among the children aged 24-59 months (N=210).

Variable	Median	Mean	SD	Min.	Max.
DDS	4.00	3.78	0.975	1	7

Where: DDS = dietary diversity score SD = standard deviation Min. = minimum Max. = maximum

**Table 2:** Distribution of the minimum dietary diversity and food group consumption among the children aged 24-59 months (N=210).

Variable	n	%
Minimum dietary diversity		
Low (<5 food groups per day)	165	78.6
Adequate (>5 food groups per day)	45	21.4
Food group	n	%
Grains, white roots and tubers and plantains		
Consumed	210	100
Not consumed	0	0
Pulses (beans, peas and lentils)		
Consumed	112	53.3
Not consumed	98	46.7
Nuts and seeds		
Consumed	13	6.2
Not consumed	197	93.8
Dairy		
Consumed	23	11
Not consumed	187	89
Meat, poultry and fish		
Consumed	172	81.9
Not consumed	38	18.1
Eggs		
Consumed	138	65.7
Not consumed	72	34.3
Dark green leafy vegetables		
Consumed	40	19
Not consumed	170	81
Other vitamin A-rich fruits and vegetables		
Consumed	29	13.8
Not consumed	181	86.2
Other vegetables		
Consumed	39	18.6
Not consumed	171	81.4
Other fruits		
Consumed	17	8.1
Not consumed	193	91.9

Table 3 presents the child and family characteristics. Most children were aged 36–59 months (69%), whereas both sexes showed similar proportions (50%). The mothers of the children in this study were mostly 19–35 years old (85.7%), had an educational level higher than primary school (68.1%) and were housewives (92.4%). The majority of the children had fathers who had educational levels higher than primary school (60.2%) and who worked as labourers (84.8%). Most children came from families with an income level below or equal to Rp 2,710,000.00 (regional minimum wage) (98.1%), a high expenditure for food (53.8%), a high expenditure for non-food (51.9%), a high total expenditure (52.4%), a family size of ≤4 persons (77.6%) and a maternal parity of ≤2 (74.8%).

**Table 3:** Child and family characteristics among the children aged 24–59 months (N=210).

Variable	n	%
Child’s age		
24–35 months	65	31
36–59 months	145	69
Child’s sex		
Male	105	50
Female	105	50
Mother’s age		
19–35 years	180	85.7
>36 years	30	14.3
Mother’s educational level		
Primary school and below	67	31.9
Higher than primary school	143	68.1
Father’s educational level		
Primary school and below	84	40.0
Higher than primary school	126	60.0
Mother’s occupation		
Housewife	194	92.4
Employed	16	7.6
Father’s occupation		
Labourer	178	84.8
Non-labourer	32	15.2
Family income		
≤Rp 2,710,000.00 (regional minimum wage)	206	98.1
>Rp 2,710,000.00 (regional minimum wage)	4	1.9
Family expenditure for food		
Low (expenditure < median [Rp 150,000.00])	97	46.2
High (expenditure ≥ median [Rp 150,000.00])	113	53.8
Family expenditure for non-food		
Low (expenditure < median [Rp 315,000.00])	101	48.1
High (expenditure ≥ median [Rp 315,000.00])	109	51.9
Total family expenditure		
Low (expenditure < median [Rp 460,000.00])	100	47.6
High (expenditure ≥ median [Rp 460,000.00])	110	52.4
Family size		
>4 persons	47	22.4
≤4 persons	163	77.6
Maternal parity		
≥3	53	25.2
≤2	157	74.8

The association of the child and family characteristics with the MDD is presented in Table 4. Family size (P=0.041) and maternal parity (P=0.038) were significantly associated with the MDD of the children (P<0.05). Compared with the children from families consisting of ≤4 persons, the children with a family size of >4 persons had 2.7 higher odds of having a low MDD. The children of mothers with a parity of ≥3 had 2.59 higher odds of having a low MDD than the children of mothers with a parity of ≤2. The study found no significant association of the child’s age, child’s sex, mother’s age, mother’s and father’s educational levels, mother’s and father’s occupation, family expenditure for food, family expenditure for non-food and total family expenditure with the MDD of the children.

**Table 4:** Association of the child and family characteristics with the MDD of the children aged 24–59 months (N=210).

Variable	Low MDD		Adequate MDD		Total		P-value	OR	95% CI
	n	%	n	%	n	%			
Child’s age									
24–35 months	46	70.8	19	29.2	65	100	0.065	0.529	0.267-1.047
36–59 months	119	82.1	26	17.9	145	100			
Child’s sex									
Male	82	78.1	23	21.9	105	100	0.866	0.945	0.489-1.827
Female	83	79.0	22	21.0	105	100			
Mother’s age									
19–35 years	138	76.7	42	23.3	180	100	0.159	0.365	0.105-1.264
>36 years	27	90	3	10	30	100			
Mother’s educational level									
Primary school and below	55	82.1	12	17.9	67	100	0.395	1.375	0.659-2.870
Higher than primary school	110	76.9	33	23.1	143	100			
Father’s educational level									
Primary school and below	70	83.3	14	16.7	84	100	0.170	1.632	0.808-3.294
Higher than primary school	95	75.4	31	24.6	126	100			
Mother’s occupation									
Housewife	151	77.8	43	22.2	194	100	0.531	0.502	0.110-2.293
Employed	14	87.5	2	12.5	16	100			
Father’s occupation									
Labourer	140	78.7	38	21.3	178	100	1.000	1.032	0.415-2.567
Non-labourer	25	78.1	7	21.9	32	100			
Family expenditure for food									
Low	77	79.4	20	20.6	97	100	0.791	1.094	0.564-2.122
High	88	77.9	25	22.1	113	100			
Family expenditure for non-food									
Low	80	79.2	21	20.8	101	100	0.868	1.076	0.556-2.082
High	85	78	24	22	109	100			
Total family expenditure									
Low	80	80	20	20	100	100	0.630	1.186	0.607-2.282
High	85	77.3	25	22.7	110	100			
Family size									
>4 persons	42	89.4	5	10.6	47	100	0.041^*^	2.732	1.012-7.377
≤4 persons	123	75.5	40	24.5	163	100			
Maternal parity									
≥3	47	88.7	6	11.3	53	100	0.038^*^	2.589	1.028-6.520
≤2	118	75.2	39	24.8	157	100			

* Significant at P<0.05.

Where: MDD = minimum dietary diversity OR = odds ratio CI = confidence interval

The Spearman rank correlation test revealed that electricity expenditure and weekly expenditure for mobile phone credit had a negative correlation with the DDS of the children (Table 5). This study found no correlation of the DDS with the child’s age, birth order in the family, mother’s age during pregnancy, number of nuclear family members, number of residents in the house and household expenditure (food, cigarette, cooking fuel, gasoline, education, healthcare, toiletries, loan, total non-food and total expenditures).

**Table 5:** Correlation of the child and family characteristics with the dietary diversity score of the children aged 24–59 months (N=210).

Variable	P-value	Correlation coefficient (r) with the dietary diversity score
Child’s age	0.169	-0.095
Birth order in the family	0.905	-0.008
Mother’s age during pregnancy	0.558	-0.041
Number of nuclear family members	0.834	-0.015
Number of residents in the house	0.569	-0.040
Food expenditure (weekly)	0.642	-0.032
Electricity expenditure (monthly)	0.000	-0.255^**^
Cigarette expenditure (weekly)	0.751	0.022
Mobile phone credit expenditure (weekly)	0.038	-0.143^*^
Cooking fuel expenditure (weekly)	0.356	0.064
Gasoline expenditure (weekly)	0.371	0.062
Education expenditure (monthly)	0.768	0.020

* Significant at P<0.05.

** Significant at P<0.01. Healthcare expenditure is blank due to the national health insurance programme by the Social Security Administrator for Health (Badan Penyelenggara Jaminan Sosial).

## Discussion

A diverse diet is essential for children under 5 years old to ensure adequate nutrient intake that supports growth and development. However, the mean DDS of the children aged 24–59 months in the current study was 3.78 out of 10 food groups. It is lower than the recommended MDD of at least 5 food groups each day.^15^ The proportion of children with a low MDD (78.6%) in Karangkamulyan Village is larger than that in South Africa (61%), Indonesia (42.9%) and Bogor City (62.5%),^7,9,18^ but smaller than that in Ethiopia (91.5%).^4^ The staple food group (consumed by 100% of the children) and the meat, poultry and fish food group (consumed by 81.9% of the children) were the most consumed food groups in the current study. Conversely, the least consumed food group was nuts and seeds (6.2%). The diet of the children in our study is similar to the general diet pattern in Indonesia, which is dominated by staple food and animal-sourced protein, while the intake of other food groups is low.^19^

In this study, a larger family size (>4 persons) elevated the risk of having a low MDD compared with a smaller family size (<4 persons). Similarly, Abdu and Mekonnen reported that family size was associated with children’s DD.^12^ One possible reason is that having more children in a household with a restricted income will strain the already scant resources allocated to various competing needs.^20^ The family will also encounter economic inadequacy to fulfil the needs of the family as their size increases. Therefore, they will focus on meeting their daily needs rather than on ensuring the quality of their diet.^21^

This study showed that the children of mothers with a higher parity (>3) had a higher risk of a low MDD than the children of mothers with a lower parity (<2). This result is in line with the report by Tegegne et al. that maternal parity was significantly associated with the MDD of children.^13^ A plausible reason for this is that mothers with a higher parity probably have more young children. Due to limited available care resources, children might compete for them.^22^ Mothers with a lower parity may also have greater motivation and commitment to properly care for and feed their children.^13^ However, the child’s sex, mother’s age, parents’ educational level and parents’ occupation were not significantly associated with the MDD of the children in this study (P>0.05).

The current study revealed a negative correlation between electricity expenditure and the DDS among the children aged 24–59 months. Almost all of the households in our study had a low income. Restricted financial resources in low-income households make the food budget compete with other essential needs. Utility bills, including electricity, are fixed costs, whereas the cost of food is considered more flexible. Consequently, low-income families might compromise food costs by purchasing cheaper and less healthy food, which might include less diverse food.^23^ Expenses for electricity, lighting, gas and housing might be prioritised over food spending.^24^ Electricity remains the largest energy source in Indonesia.^25^ In Banten Province, 100% of rural areas are already electrified.^26^ Access to modern energy, including electricity, is one of the main determinants of energy spending, especially for households with low income and residing in rural areas. A study in Sri Lanka showed a 20% decline in food spending in households consisting of four adults with access to electricity compared with households without electricity.^27^

In our study, expenditure for mobile phone credit negatively influenced the DDS. In Indonesia, the development of information and communication technology caused a shift of usage from fixed-line telephones to mobile phones. In 2020, 90.75% of Indonesian households owned at least one mobile phone. The majority of these mobile phone users were pre-paid subscribers. Therefore, mobile phone credit is a routine expense. This credit is used not solely to maintain communication but also to purchase quota for accessing the internet, which has various functions. Moreover, during the coronavirus disease pandemic, mobile phone credit expenses increased to support school activities and work from home. Hence, mobile phone credit expenditure might become an essential need that could reduce spending on diverse food for children under 5 years old. According to Breunig and McCarthy, telecommunications expenditure might act like other essential expenditures, such as those for food.^28^ A study conducted by The Office of Communications in the United Kingdom showed that more than a million households reduced spending for food and clothes to pay their telecommunications bills during the pandemic in 2020.^29^

The low mean DDS and small proportion of children with an adequate MDD suggest the need for interventions to improve parents’ knowledge and practice in complementing their children’s DD. Interventions to increase accessibility to diverse foods also need to be undertaken to increase success. The number of family members and maternal parity as the factors associated with the MDD emphasise the need to encourage the implementation of family planning programmes. To our knowledge, this study is the first to reveal the correlation of electricity expenditure and mobile phone credit expenditure with the DDS of children aged 24–59 months. These results show the importance of multisectoral collaboration in improving the dietary quality among children.

However, some limitations of this study need to be considered. First, the cross-sectional design of this study limits the ability to evaluate the causal effect between the independent and dependent variables. Second, the study was conducted within one village. Therefore, the findings cannot be generalised to the national level. Despite these limitations, our study adds useful information about the factors associated with the DD of children aged 24–59 months in Indonesia, which can be used as a basis for programmes and policymaking to reduce the prevalence of stunting and other undernutrition conditions.

## Conclusion

This study revealed that the DD among children aged 24–59 months in Karangkamulyan Village was generally low, and the majority did not meet the MDD. Family characteristics, especially larger family size and higher maternal parity, were significantly associated with a low MDD. Our study also found that electricity expenditure and mobile phone credit expenditure negatively correlated with the DDS. These findings portray the importance of considering various factors that impact children’s nutrition. Therefore, collaboration among various stakeholders is crucial to improve the dietary quality and nutritional status among children. Further research is also needed to evaluate the factors associated with the DD of children aged 24–59 months in Indonesia at different locations and with different community characteristics.

## Data sharing statement

The data used in this study are not currently publicly available due to participant privacy and consent.

**How does this paper make a difference in general practice?**

- This paper presents that children’s dietary diversity correlates with non-food expenditure.
- The correlations show the importance of multisectoral collaboration in improving the dietary quality for children.
- This study portrays the descriptive data of dietary diversity and food group consumption among children in Indonesia, given that these data on children in Southeast Asia remain scarce. The findings show one of the dietary quality dimensions among the population.

## References

[1] Preedy VR, Hunter LA, Patel VB (2013). Diet Quality: An Evidence-Based Approach.. Vol 2..

[2] Food and Agriculture Organization of the United Nations (2010). Guidelines for Measuring Household and Individual Dietary Diversity..

[3] Food and Agriculture Organization of the United Nations (2016). Minimum Dietary Diversity for Women: A Guide to Measurement..

[4] Woldegebriel AG, Desta AA, Gebreegziabiher G, Berhe AA, Ajemu KF, Woldearegay TW (2020). Dietary diversity and associated factors among children aged 6-59 months in Ethiopia: analysis of Ethiopian Demographic and Health Survey 2016 (EDHS 2016).. IntJPediatr..

[5] Sie A, Tapsoba C, Dah C (2018). Dietary diversity and nutritional status among children in rural Burkina Faso.. Int Health..

[6] Harper A, Goudge J, Chirwa E, Rothberg A, Sambu W, Mall S (2022). Dietary diversity, food insecurity and the double burden of malnutrition among children, adolescents and adults in South Africa: findings from a national survey.. Front Public Health..

[7] Modjadji P, Molokwane D, Ukegbu PO (2020). Dietary diversity and nutritional status of preschool children in North West Province, South Africa: a cross sectional study.. Children (Basel)..

[8] UNICEF (1990). Strategy for Improved Nutrition of Children and Women in Developing Countries..

[9] Utami NH, Mubasyiroh R (2020). Dietary diversity and its association with nutritional status among children under five years-old: results of Indonesian Food Consumption Survey (IFCS) [Keragaman makanan dan hubungannya dengan status gizi balita: analisis Survei Konsumsi Makanan Individu].. Gizi Indones..

[10] Beyene M, Worku AG, Wassie MM (2015). Dietary diversity, meal frequency and associated factors among infant and young children in Northwest Ethiopia: a cross-sectional study.. BMC Public Health..

[11] Rah JH, Akhter N, Semba RD (2010). Low dietary diversity is a predictor of child stunting in rural Bangladesh.. Eur J Clin Nutr..

[12] Abdu AO, Mekonnen BA (2019). Determinants of dietary adequacy among school age children in Guraghe Zone, Southern Ethiopia.. Int J Public Heal Sci..

[13] Tegegne M, Sileshi S, Benti T, Teshome M, Woldie H (2017). Factors associated with minimal meal frequency and dietary diversity practices among infants and young children in the predominantly agrarian society of Bale zone, Southeast Ethiopia: a community based cross sectional study.. Arch Public Health..

[14] Lwanga SK, Lemeshow S (1991). Sample Size Determination in Health Studies: A Practical Manual..

[15] Food and Agriculture Organization of the United Nations (2021). Minimum Dietary Diversity for Women: An Updated Guide for Measurement: From Collection to Action..

[16] Diop L, Becquey E, Turowska Z, Huybregts L, Ruel MT, Gelli A (2021). Standard minimum dietary diversity indicators for women or infants and young children are good predictors of adequate micronutrient intakes in 24-59-month-old children and their nonpregnant nonbreastfeeding mothers in rural Burkina Faso.. J Nutr..

[17] Caswell BL, Talegawkar SA, Siamusantu W, West KP, Palmer AC (2018). A 10-food group dietary diversity score outperforms a 7-food group score in characterizing seasonal variability and micronutrient adequacy in rural Zambian Children.. J Nutr..

[18] Trisasmita L, Sudiarti T, Sartika RAD, Setiarini A (2020). Identification of dietary diversity associated with stunting in Indonesia.. Malays J Nutr..

[19] Food and Agriculture Organization of the United Nations, Government of Indonesia, World Food Programme (2017). Food Security Monitoring Bulletin: Indonesia, Special Focus: Food Security in 100 Districts Prioritized for Reduction of Stunting..

[20] Huluka AT, Wondimagegnhu BA, Yildiz F (2019). Determinants of household dietary diversity in the Yayo biosphere reserve of Ethiopia: an empirical analysis using sustainable livelihood framework.. Cogent FoodAgric..

[21] Endalifer ML, Andargie G, Mohammed B, Endalifer BL (2021). Factors associated with dietary diversity among adolescents in Woldia, Northeast Ethiopia.. BMC Nutr..

[22] Amugsi DA, Dimbuene ZT, Kimani-Murage EW (2020). Socio-demographic factors associated with normal linear growth among pre-school children living in better-off households: A multi-country analysis of nationally representative data.. PLoS One..

[23] Ward PR, Verity F, Carter P, Tsourtos G, Coveney J, Wong KC (2013). Food stress in Adelaide: the relationship between low income and the affordability of healthy food.. J Environ Public Health..

[24] More J (2021). Infant, Child and Adolescent Nutrition: A Practical Handbook..

[25] Ministry of Energy and Mineral Resources of Republic of Indonesia (2020). Handbook of Energy & Economy Statistics of Indonesia 2020..

[26] Secretariat of the Directorate General of Electricity Ministry of Energy and Mineral Resources (2021). Statistik Ketenagalistrikan 2020..

[27] Jayasinghe M (2022). Household electrification, food consumption and welfare nexus in Sri Lanka: an intertemporal analysis.. J Asia Pacific Econ..

[28] Breunig R, McCarthy O (2020). Household telecommunications expenditure in Australia.. Telecomm Policy..

[29] The Office of Communications United Kingdom (2020). 4.7 Million UK Homes Have Struggled to Afford Their Telecoms Bills This Year.. https://www.ofcom.org.uk/news-centre/2020/4.7-million-uk-homes-have-struggled-to-afford-their-telecoms-bills-this-year.

